# Longitudinal Association of Telomere Attrition with the Effects of Antihypertensive Treatment and Blood Pressure Lowering

**DOI:** 10.14336/AD.2019.0721

**Published:** 2019-07-21

**Authors:** Shuyuan Zhang, Rongxia Li, Yunyun Yang, Yu Chen, Shujun Yang, Jian Li, Cunjin Wu, Tao Kong, Tianlong Liu, Jun Cai, Li Fu, Yanan Zhao, Rutai Hui, Weili Zhang

**Affiliations:** ^1^State Key Laboratory of Cardiovascular Disease, FuWai Hospital, National Center for Cardiovascular Diseases, Peking Union Medical College & Chinese Academy of Medical Sciences, Beijing, China; ^2 ^The Second Hospital of Tianjin Medical University, Tianjin, China; ^3 ^Hypertension Centre, FuWai Hospital, National Center for Cardiovascular Diseases, Peking Union Medical College & Chinese Academy of Medical Sciences, Beijing, China; ^4^Benxi Railway Hospital, Liaoning, China

**Keywords:** leukocytes telomere length, telomere attrition, blood pressure lowering, antihypertensive therapy

## Abstract

Leukocytes telomere length has been associated with hypertension, but, whether longitudinal telomeres change could serve as a useful predictive tool in hypertension remains uncertain. This study aimed to examine the longitudinal trajectory of leukocytes telomere length in a population-based prospective study of 1,108 individuals with hypertension. Leukocytes telomere length were measured at baseline and again after a median 2.2 (range 1.5-2.4) years of follow-up. Age as an independent predictor was inversely associated with baseline telomeres and follow-up telomeres. Annual telomere attrition rate was calculated as (follow-up telomeres-baseline telomeres)/follow-up years, and participants were categorized into the shorten and the lengthen groups. Results showed that telomere lengthening was significantly correlated with decreased systolic blood pressure (SBP) (*β*=-3.28;* P*=0.02) and pulse pressure (PP) (*β*=-2.53; *P*=0.02), and the differences were respectively -3.3 mmHg (95%CI, -6.2 to -0.3; *P*=0.03) in ∆SBP and -2.4 mmHg (95%CI, -4.9 to -0.1; *P*=0.04) in ∆PP between two groups after adjustment for vascular risk factors and baseline blood pressures. When stratified by age and gender, the correlations were observed in women and patients ≤60 years. Furthermore, among patients using calcium channel blocker (CCB) and angiotensin receptor blocker (ARB), those with telomeres lengthening showed a significantly lower level of ∆SBP and ∆PP. There was no correlation between telomere attrition and incidence of cardiovascular events. Our data indicated that increased telomere length of leukocytes was associated with decreased SBP and PP, particularly for patients who received CCB and ARB, supporting that telomere attrition may provide new sight in clinical intervention for hypertension.

Telomeres are tandem repeat nucleotides sequence (TTAGGG)_n _at the end of linear chromosomes which form a protective cap and maintain the chromosomal stability [1]. Telomere length is genetically determined at birth and shortened gradually with aging [2,3]. Attrition rate of telomere length reflects the cumulative lifetime burden of genetic factors and environmental stressors of a person more precisely than chronological age, and hence, it has been an appealing research target [4]. Emerging evidence has demonstrated that age-dependent telomere length shortening in circulating leukocytes is correlated with higher risk of atherosclerosis as well as cardiovascular diseases [5-8], and leukocytes telomere dynamics contribute to the age-related process of vascular damage and cardiovascular mortality [9-11]. To date, studies have not drawn convincing conclusions with respect to the relationship between telomere length and hypertension.

Aging is a major risk factor for hypertension. Telomere length has been reported to inversely associate with increased systolic blood pressure (SBP) and pulse pressure (PP), which is familial [12, 13]. Several epidemiology studies showed that mean leukocyte telomere length is shorter in hypertensive than in normotensive subjects and contributes to the risk of developing hypertension and future cardiovascular diseases [14-16], whereas not found in other studies [17]. The Framingham heart study has demonstrated that association between hypertension and leukocyte telomere length is attributable to insulin resistance [18]. The inconsistency may be partly explained by the limitation of a single measurement of baseline telomere length in these studies, as well as the difference in geography and ethnicity. Whether the rate of telomere attrition could serve as a useful predicting tool in hypertensive patients requires further data on longitudinal change of telomere length during the follow-up.

In addition, antihypertensive drugs may affect cell senescence and telomere length. Data from the Framingham heart study showed that shortened leukocyte telomere length is more frequently present in hypertensive patients with a higher plasma renin-angiotensin ratio [19], and treatment with an angiotensin II receptor antagonist can prevent telomere length shortening in spontaneously hypertensive rats through attenuating the process of aging [20], which indicates a close link between telomere length and the renin-angiotensin-aldosterone system (RAAS). It has been reported that the individual-level difference in blood pressure response to antihypertensive drugs can be induced in large part by differences in plasma renin activity [21]. Moreover, the calcium channel blocker (CCB) is also shown to affect telomere length through an endothelial nitric oxide synthase (eNOS)-dependent anti-senescence effect in human endothelial cells [22]. However, there is lack of data whether change of telomere length in patients with hypertension is related to the heterogeneity of blood pressure response to antihypertensive therapy.

Therefore, in this study, we aimed to investigate longitudinal change of leukocytes telomere length in a prospective population-based study of 1,108 individuals with hypertension who received antihypertensive therapy, and further assess the relationship between BP lowering and telomere attrition rate, which may provide new sight on telomere biology in hypertension and antihypertensive intervention.

## MATERIALS AND METHODS

### Study Design and Participants

This prospective community-based study was conducted in the BenXi County, Liaoning Province, in the northern region in China. A multistage cluster sampling method was used to select a representative sample of urban community residents aged 35 to 75 years. First, three communities were randomly selected from a total of six residential communities in the BenXi County. Secondly, 12 districts were randomly selected from a total of 24 districts in this three residential communities. A total of 13,000 subjects (7,540 men and 5,460 women) completed the survey with a response rate of 85.1%. Among them, 3,671 participants with primary hypertension were identified and recruited in this cohort. Subjects were excluded from the study when they had any known diseases including heart failure, renal failure, valvular heart disease, or severe debilitating chronic illness (cancer or hepatic diseases). History of possible secondary hypertension was evaluated using a standardized questionnaire assessment when the patients were surveyed, including history of renal diseases, sleep apnoea, symptoms suggestive of thyroid disease or hyperparathyroidism, signs of Cushing’s disease or acromegaly, young onset of stage 2 or 3 hypertension (<40 years), or sudden development of hypertension or rapidly worsening BP in older patients. Those with known secondary hypertension (e.g. renal artery stenosis, chronic renal insufficiency, or endocrine origin) diagnosed by imaging techniques and humoral measurements, were excluded. In addition, the apparent treatment-resistant hypertensions were also excluded which used the following definitions: uncontrolled BP (≥140/90 mm Hg) despite antihypertensive regimen of at least 3 different antihypertensive drugs including diuretics, or controlled BP (<140/90 mm Hg) while being received with ≥4 antihypertensive drugs [23].

Among the 3,671 hypertensive patients recruited in this cohort, 1,382 subjects provided blood samples at baseline. No significant differences in characteristics were observed between total recruited patients and those having blood samples, except that those providing blood samples had a little higher level of SBP and medical history of cardiovascular diseases and diabetes mellitus (Supplemental Table S1). This cohort was prospectively followed up at a 2.5-year period, blood samples were collected again in 2016, and 1,197 patients provided for the second time. A total of 185 patients were not reached due to immigration, in addition, 89 blood samples were excluded due to insufficient quality. Finally, 1,108 patients available with blood samples both at baseline and at follow-up, were included in the analysis for telomeres change, as shown in the flowchart (Supplementary Fig. 1). There were no significant differences between patients with and without the follow-up blood samples (Supplemental Table S2).

This study protocol was reviewed and approved by the Ethics Committee of FuWai hospital and local hospitals. All participants gave their written informed consent.

### Collection of Data at Baseline

Each enrolled participant was interviewed and completed a standardized questionnaire assessment at baseline survey that included demographic characteristics (e.g., age, gender, education levels), medical history, drugs used for hypertension, and lifestyle behaviors (e.g., smoking, drinking, and exercise). Body weight and height were measured by trained nurses, and body mass index (BMI) was calculated as weight in kilograms divided by the square of height in meters. Waist circumference was measured on standing subjects with a soft tape midway between the lowest rib and the iliac crest, and hip circumference was measured over the widest part of the gluteal region.

BP was measured by trained nurses with a validated oscillometric BP monitor with appropriately sized arm cuffs (regular adult, large, or small). All participants were advised to avoid alcohol, cigarette smoking, coffee/tea, and exercise for at least 30 minutes before their BP measurement. The average of three readings in a sitting position after at least 5 minutes of rest, recorded at least 5 minutes apart, was obtained for analysis. Hypertension was defined as systolic BP ≥140 mm Hg and/or diastolic BP ≥90 mm Hg, and/or receiving antihypertensive drugs, and/or history of hypertension, according to the 2010 Chinese Guidelines for the Management of Hypertension [24]. The stages of hypertension at baseline were classified into 4 groups: the controlled BP, stage 1, 2, and 3 hypertension, in which the controlled BP was defined as BP<140/90 mm Hg, stage 1 as SBP 140-159 mm Hg and/or DBP 90-99 mm Hg, stage 2 as SBP 160-179 mm Hg and/or DBP 100-109 mm Hg, and stage 3 as SBP ≥180 mm Hg, and/or DBP≥110 mm Hg.

### Blood samples and measurement of biochemical parameters

Blood samples were collected from the antecubital vein after an overnight fast at baseline. This study included the hypertensive patients who either never received antihypertensive therapy at the first interview, or already received antihypertensive drug treatment. For the former participants, we collected their blood samples before hypertensive drug intervention. For the patients on previous antihypertensive drug therapy, they were required to receive the antihypertensive agents provided by the study at no cost, including calcium channel blocker (CCB), angiotensin receptor blocker (ARB), angiotensin converting enzyme inhibitor (ACEI), or thiazide-type diuretics, unless intolerance was reported or there was a compelling indication for other drugs. In this case, the blood samples were collected at two weeks after the enrollment. We compared the baseline characteristics between included patients with and without receiving antihypertensive treatment at recruitment, and the results showed no significant differences between the two groups, except that those never receiving antihypertensive drugs had a lower rate of history of cardiovascular diseases. Moreover, there were no remarkable differences in the baseline telomere length of leukocytes and annual telomere attrition rate during the follow-up between the two groups (Supplemental Table S3).

Blood serum was separated on-site, then transported on dry ice to Beijing center laboratory, and stored at -80°C until measurement. Concentrations of total cholesterol (TC), high-density lipoprotein cholesterol (HDL-C), low-density lipoprotein cholesterol (LDL-C), triglycerides (TG), and fasting glucose were measured by an automatic analyzer (Hitachi 7060, Hitachi, Tokyo, Japan). All measurements were taken at Beijing FuWai Clinical Laboratory qualified by the Centers for Disease Control and Prevention.

### Follow-up and Outcome Assessment

Patients were followed up by face-to-face interviews by trained physicians from May to November in 2016. Information on anthropometric measurements, physical examination, lifestyle behaviors, and drugs used for hypertension were updated via structured questionnaires during the follow-up survey. Participants underwent routine assessment of their blood pressure using the same standardized protocol as the baseline. Blood samples after a 12-hour overnight fast were collected again for measuring biochemical parameters and leukocytes telomere length. The main outcome was defined as a composite of myocardial infarction (MI), stroke (ischemic or hemorrhagic, fatal or nonfatal), hospitalization for unstable angina or acute decompensated heart failure, coronary revascularization, and deaths from cardio-vascular causes. The endpoints were ascertained by local physicians primarily through self-reports and review of medical records, and clinical medical records and imaging evidence were required to support all diagnosis. Deaths were reported by family members, work associates and/or obtained from death certificates and medical records. Definition of the endpoints were presented in the Supplementary materials.

### Measurement of Leukocytes Telomere Length

Genomic DNA was isolated from peripheral blood leukocytes according to standard procedures using MiniBEST Universal Genomic DNA Extraction Kit (Takara Biomedical Technology Co., Ltd., Dalian, China). Relative mean telomere length of leukocytes was determined by a quantitative real-time PCR method which compares telomere repeat copy number (T) to single-copy gene copy number β-globin (S) (T/S ratio) as described previously [25, 26]. In brief, each sample was measured in triplicates on an ABI 7500 Real-Time PCR System (Applied Biosystems) and the mean relative T/S ratio was calculated. A reference calibrator sample was included with each measurement to control inter-assay variability, and the average inter-plate coefficients of variability for the telomere and β-globin assays were <5.0%. A standard curve was also examined by using serially diluted reference DNA (1.56-100 ng; 2-fold dilution; seven points) with good linearity (R^2^> 0.97) for both the telomere and the β-globin measurement. The primers were as the following: for telomeres, forward 5’-CGGTTTGTTTGGGTTTGGGTTTGGGTTTGGGTTT-GGGTT-3’ and reverse 5’-GGCTTGCCTTACCCTTA- CCCTTACCCTTACCCTTACCCT-3’; for β-globin measurement, forward 5’-GCTTCTGACACAACTG- TGTTCACTAGC-3’ and reverse 5’-CACCAACTTC- ATCCACGTTCACC-3’. In this study, 20% of samples were randomly chosen to test the reproducibility of measurements.

### Statistical Analysis

Clinical characteristics of participants were compared between groups by the chi-square test for categorical variables (expressed as numbers [percentages]) and the* t* test for quantitative variables (expressed as mean ± standard difference [SD]). For triglycerides and relative mean telomere length of leukocytes, the non-parametric Mann-Whitney *U* test was used due to their sewed distribution. In this study, values of telomere length were logarithm (Lg) transformed so as to reach a normal distribution for analyzing the relation between telomere attrition and blood pressure lowering. Annual telomere attrition rate was calculated using the formula: (follow-up telomere length-baseline telomere length)/follow-up years. All participants were categorized into two groups, of those who experienced telomere length shortening (annual rate of telomere attrition <0) were categorized as “shorten group”, and those who experienced an increase of telomere length (annual rate of telomere attrition >0) as “lengthen group”. Here, according to difference in means of telomere length (Lg-transformed) between the shorten (mean, 0.24; SD 0.19) and the lengthen group (mean, 0.07; SD, 0.21), the power would achieve >98% to detect a difference in blood pressure lowering with a two-sided α level of 0.05 in 1,108 participants by using the PASS software program (www.ncss.com).

Linear regression analysis was used to examine the relation between annual telomere attrition rate and baseline telomere length and age. The correlations of annual telomere attrition rate with SBP change (ΔSBP), DBP change (ΔDBP) and pulse pressure change (ΔPP) (ΔPP=ΔSBP-ΔDBP) were examined by multivariate linear regression models which first adjusted for age, gender, medical history, baseline features of smoking status (current, former, or never), alcohol intake (current, former, or never), BMI, waist-to-hip, the stage of baseline blood pressures, fasting glucose, TC, triglycerides, HDL-C, LDL-C, and telomere length (model I), and then further adjusted for changes in BMI, waist-to-hip, fasting glucose, total cholesterol, triglycerides, HDL-C, and LDL-C from 2014 to 2016 during the follow-up. Generalized linear regression model was used to compare the differences in blood pressure change between the shorten and the lengthen group which adjusted for age, gender and covariates mentioned above. The effects of antihypertensive drug treatment on the correlation of annual telomere attrition rate with blood pressure lowering were examined in further stratified analysis by age and gender.

The Cox proportional hazards regression model was used to examine the association between annual telomere attrition rate and cardiovascular outcome events. Person-years of follow-up started from the date of recruitment until the occurring date of cardiovascular outcomes, death, or the end of follow-up (November 30, 2016), whichever came first. A two-tailed probability value of ≤0.05 was considered significant. Analyses were performed with SPSS Statistics 20.0 (SPSS Inc, Chicago, USA).

## RESULTS

### Clinical Characteristics of Hypertensive Patients

A total of 1,108 hypertensive patients (age 31 to 89 years) with blood samples both at baseline and at follow-up were included in this study for assessing the telomeres change, as shown in the flowchart (Supplementary Fig. 1). The average age of patients was 61.7 (SD=9.7) years and men accounted for 38.8%. In this study, the values of baseline telomere length and follow-up telomere length of leukocytes showed a skewed distribution, and after further logarithmic transformation, they showed a normal distribution for analysis (Supplementary Fig. 2). Age as an independent predictor was inversely associated with both baseline telomere length (*β*= -0.14;* P*<0.001) and follow-up telomere length (*β*= -0.16; *P*<0.001) (Supplementary Fig. 3).

**Figure 1.**
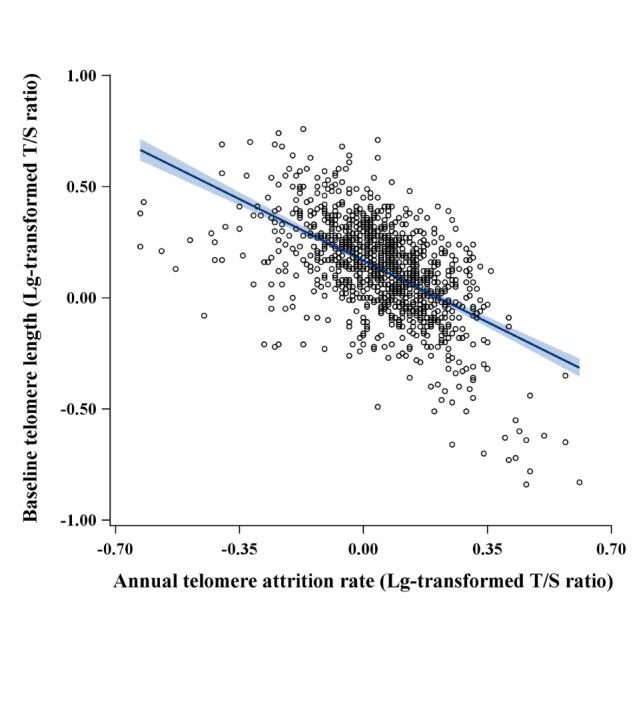
Inverse correlation between annual telomere attrition rate and baseline telomere length of leukocytes. The leukocyte telomere length of all participants was plotted as Lg-transformed T/S ratio. Annual telomere attrition rate was calculated by the equation: (follow-up telomere length- baseline telomere length)/follow-up years. The correlation coefficient *β *was -0.52 and *R^2^* was 0.27 (*P*<0.001).

All studied participants were divided into two groups depending on annual rate of telomere attrition, one is the shorten group (n=386) who had a decrease of telomere length, and the other is the lengthen group (n=722) who had an increase of telomere length (Supplemental Figure S4). The clinical characteristics at baseline were shown in Table 1, and there were no significant differences in BMI, blood pressure, lipid profiles, fasting glucose, smoking status, alcohol intake, and medical history of cardiovascular diseases. Of note, patients who experienced telomere shortening at follow-up were more likely to have a longer baseline telomere length (median, 1.8; interquartile range, 1.4-2.3), and in contrast, those who experienced telomere lengthening at follow-up had a shorter baseline telomere length (median, 1.2; interquartile range, 0.9-1.6). There was a significantly inverse correlation between annual telomere attrition rate and baseline telomere length (*β*=-0.52; *P*<0.001) (Fig. 1). Aging was an independent predictor for telomere length attrition rate after adjustment for baseline telomere length (Supplementary Table 4).

Considering that the stage of baseline blood pressures might affect the relation between telomere attrition rate and blood pressure lowering, we also compared the characteristics and annual telomere attrition rate for hypertensive patients of different stages (Supplemental Table S5). Patients at stage 3 hypertension were more likely to be older, and had a higher level of serum glucose compared with those having the controlled BP (<140/90 mmHg). Moreover, the baseline leukocytes telomere length of patients at stage 1, 2 and 3 hypertension were significantly shorter than that of those having the controlled BP (All *P*<0.05). There were no remarkable differences among the stages of hypertension for other traditional vascular risk factors including BMI, lipid profiles, smoking status, alcohol intake, and medical history of cardiovascular diseases as well as diabetes mellitus. In the further analysis of association between telomere attrition rate and blood pressure lowering, we further adjusted for the stage of baseline blood pressure as a covariate in the multiple linear regression models.

**Table 1:** Baseline characteristics of patients between the shorten and lengthen groups categorized by annual telomere attrition rate during 2014-2016.

		Annual telomere attrition rate		
Characteristics	Total (n=1,108)	Shorten group (n=386)	Lengthen group (n=722)	*P *value^†^
Age, years	61.7 ± 9.7	62.3 ± 9.6	61.4 ± 9.8	0.13
Men, No. (%)	430 (38.8%)	155 (40.2%)	275 (38.1%)	0.52
BMI, kg/m^2^	26.2 ± 3.1	26.3 ± 3.2	26.2 ± 3.1	0.85
Waist-to-hip ratio	0.90 ± 0.05	0.90 ± 0.05	0.90 ± 0.05	0.09
Systolic BP, mm Hg	160 ± 21	159 ± 21	160 ± 21	0.27
Diastolic BP, mm Hg	89 ± 12	89 ± 11	89 ± 12	0.92
Fasting serum glucose, mmol/L	6.2 ± 1.7	6.1 ± 1.5	6.3 ± 1.8	0.10
Lipids, mmol/L				
Total cholesterol	5.7 ± 1.1	5.6 ± 1.1	5.7 ± 1.1	0.41
Triglycerides	1.6 (1.1-2.3)	1.6 (1.1-2.2)	1.6 (1.1-2.4)	0.23
HDL-C	1.3 ± 0.3	1.3 ± 0.3	1.3 ± 0.3	0.40
LDL-C	3.6 ± 0.9	3.6 ± 0.9	3.6 ± 0.9	0.48
Smoking status, %				
Never	73.8	73.1	74.1	0.41
Former	7.4	8.8	6.6	
Current	18.8	18.1	19.1	
Alcohol intake, %				
Never	77.0	77.7	76.6	0.58
Former	5.1	5.7	4.7	
Current	18.0	16.6	18.7	
Medical history, %				
Diabetes mellitus	23.8	21.8	24.9	0.27
Stroke	21.1	23.1	20.1	0.25
Coronary heart disease	29.7	30.6	29.2	0.68
Antihypertensive drugs, No. (%)				
Calcium channel blocker	744 (67.1)	251 (65.0)	493 (68.3)	0.28
Angiotensin receptor blocker	590 (53.2)	192 (49.7)	398 (55.1)	0.09
ACE inhibitor	87 (7.9)	35 (9.1)	52 (7.2)	0.29
Beta-blocker	24 (2.2)	11 (2.8)	13 (1.8)	0.28
Diuretics	300 (27.1)	104 (26.9)	196 (27.1)	0.99
Leukocytes telomere length, T/S ratio				
At baseline	1.4 (1.0-1.8)	1.8 (1.4-2.3)	1.2 (0.9-1.6)	<0.001
At follow-up	1.9 (1.3-2.5)	1.1 (0.8-1.5)	2.2 (1.7-3.0)	<0.001
Telomere length, base pairs (Kb)^*^				
At baseline	6.6 (5.7-7.7)	7.5 (6.5-8.9)	6.3 (5.5-7.2)	<0.001
At follow-up	7.8 (6.3-9.4)	6.0 (5.2-6.9)	8.6 (7.4-10.4)	<0.001

Abbreviations: BMI, Body mass index; BP, blood pressure; HDL-C, high-density lipoprotein cholesterol; LDL-C, low-density lipoprotein cholesterol; ACE, angiotensin converting enzyme; T, telomere repeat copy; S, single-copy gene globin copy; Kb, kilo base pairs. Data were given as mean ± SD, number (%), or median (interquartile range). The leukocytes telomere length was expressed as T/S ratio. ^*^Base pairs of telomere length were calculated based on the equation= 3274 + 2413 × (T/S ratio)^ [55]^. ^†^*P* value was calculated by the chi-square test for categorical variables, the* t* test for continuous variables, or the Mann-Whitney *U* test for triglycerides and telomere length.

**Table 2:** Association of annual telomere attrition rate with BP change during 2014-2016.

	Annual telomere	Mode I^*^			Model II^†^		
Change in BP	attrition rate	*β*	SE	*P *value	*β*	SE	*P *value
Total (n=1,108)							
ΔSBP	Shorten (n=386)	Ref.				Ref.	
	Lengthen (n=722)	-3.20	1.35	0.02	-3.28	1.37	0.02
ΔDBP	Shorten (n=386)	Ref.			Ref.		
	Lengthen (n=722)	-0.58	0.73	0.43	-0.78	0.74	0.29
ΔPP	Shorten (n=386)	Ref.			Ref.		
	Lengthen (n=722)	-2.74	1.10	0.01	-2.53	1.11	0.02
Men (n=430)							
ΔSBP	Shorten (n=155)	Ref.			Ref.		
	Lengthen (n=275)	-2.19	2.14	0.31	-2.68	2.16	0.15
ΔDBP	Shorten (n=155)	Ref.			Ref.		
	Lengthen (n=275)	-0.78	1.20	0.52	-1.05	1.19	0.38
ΔPP	Shorten (n=155)	Ref.			Ref.		
	Lengthen (n=275)	-1.48	1.73	0.39	-1.34	1.72	0.44
Women (n=678)							
ΔSBP	Shorten (n=231)	Ref.			Ref.		
	Lengthen (n=447)	-3.91	1.73	0.02	-3.56	1.76	0.04
ΔDBP	Shorten (n=231)	Ref.			Ref.		
	Lengthen (n=447)	-0.71	0.92	0.45	-0.81	0.94	0.39
ΔPP	Shorten (n=231)	Ref.			Ref.		
	Lengthen (n=447)	-3.42	1.42	0.02	-3.25	1.44	0.03
≤60 years (n=506)							
ΔSBP	Shorten (n=169)	Ref.			Ref.		
	Lengthen (n=337)	-6.41	1.90	0.001	-6.82	1.91	<0.001
ΔDBP	Shorten (n=169)	Ref.			Ref.		
	Lengthen (n=337)	-2.37	1.10	0.03	-2.49	1.11	0.03
ΔPP	Shorten (n=169)	Ref.			Ref.		
	Lengthen (n=337)	-3.97	1.46	0.007	-4.23	1.47	0.004
>60 years (n=602)							
ΔSBP	Shorten (n=217)	Ref.			Ref.		
	Lengthen (n=385)	-1.53	2.08	0.46	-1.42	2.13	0.51
ΔDBP	Shorten (n=217)	Ref.			Ref.		
	Lengthen (n=385)	0.76	0.97	0.43	0.41	1.0	0.68
ΔPP	Shorten (n=217)	Ref.			Ref.		
	Lengthen (n=385)	-1.46	1.58	0.35	-1.74	1.77	0.33

Abbreviations: BP, blood pressure; SBP, systolic blood pressure; DBP, diastolic blood pressure; PP, pulse pressure; *β*, regression *β* coefficients; SE, standard error; Ref., reference. ^*^Model I and ^†^Model II were multiple linear regression models for analyzing the correlation between annual telomere attrition rate and BP change during 2014-2016. ^*^Model I adjusted for baseline characteristics including age, gender (except in gender-stratified analysis), BMI, waist-to-hip, smoking status, alcohol intake, medical history, the stage of baseline blood pressures, leukocytes telomere length, serum fasting glucose, total cholesterol, triglycerides, HDL-C, and LDL-C. ^†^Model II further adjusted for changes in BMI, waist-to-hip, fasting glucose, total cholesterol, triglycerides, HDL-C, and LDL-C from 2014 to 2016, besides the covariates in Model I.

**Fig 2.**
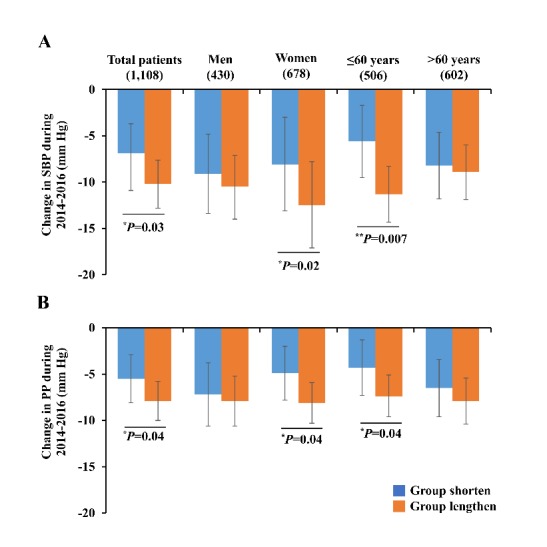
Differences of blood pressure lowering between the lengthen and the shorten groups during 2014-2016. Abbreviations: SBP, systolic blood pressure; PP, pulse pressure. ^*^P<0.05, ^**^P<0.01. *P* values and adjusted mean were calculated by generalized linear model adjustment for covariates including age, gender (except in gender-stratified analysis), smoking status, alcohol intake, medical history, the stage of baseline blood pressures, baseline telomere length, and changes in BMI, waist-to-hip, fasting glucose, TC, triglycerides, HDL-C, and LDL-C from 2014 to 2016. Error bars indicate 95%CI.

### Correlation of telomere attrition with blood pressure lowering

Compared with hypertensive patients in the shorten group who experienced telomere length shortening, those in the lengthen group showed significantly lower levels of SBP and PP during follow-up from 2014 to 2016. With the use of generalized linear regression analysis adjusting for covariates including age, gender, smoking and alcohol status, medical history, the stage of baseline blood pressures, baseline telomere length, and changes in BMI, waist-to-hip, fasting serum glucose, TC, triglycerides, HDL-C, and LDL-C during 2014-2016, the results showed that ∆SBP was -10.2 mm Hg (95%CI, -12.8 to -7.6) for the lengthen group and -6.9 mm Hg (95%CI, -10.1 to -2.9) for the shorten group (*P*=0.03) (Fig. 2A); the ∆PP was -7.9 mm Hg (95%CI, -10.0 to -5.8) for the lengthen and -5.5 mm Hg (95%CI, -8.1 to -2.9) for the shorten group (*P*=0.04) (Fig. 2B). Next, when stratified by age and gender, the differences in ∆SBP and ∆PP were more significant in women (*P_interaction_*=0.05 by gender) and patients aged≤60 years (*P_interaction_*<0.05 by age) (Supplementary Table 6). There was no significant difference in change of DBP between the lengthen and shorten group.

The correlation coefficients between annual telomere attrition rate and blood pressure lowering were further assessed by multiple linear regression analysis, and the coefficient *β* was -3.28 (*P*=0.02) for ∆SBP and -2.53 (*P*=0.02) for ∆PP, respectively (Table 2). The correlations still remained after adjustment for changes in BMI, waist-to-hip, fasting glucose, TC, triglycerides, HDL-C, and LDL-C at follow-up. Similarly, when stratified by age and gender, the correlations between annual telomere attrition rate and ∆SBP or ∆PP were observed in women and patients aged≤60 years. We also examined whether baseline telomere length could affect blood pressure lowering and found no significant correlation between baseline telomere length and change in SBP or PP (Supplemental Fig. 5).

### Association Between Telomere Attrition and the Effects of Antihypertensive Treatment

**Fig 3.**
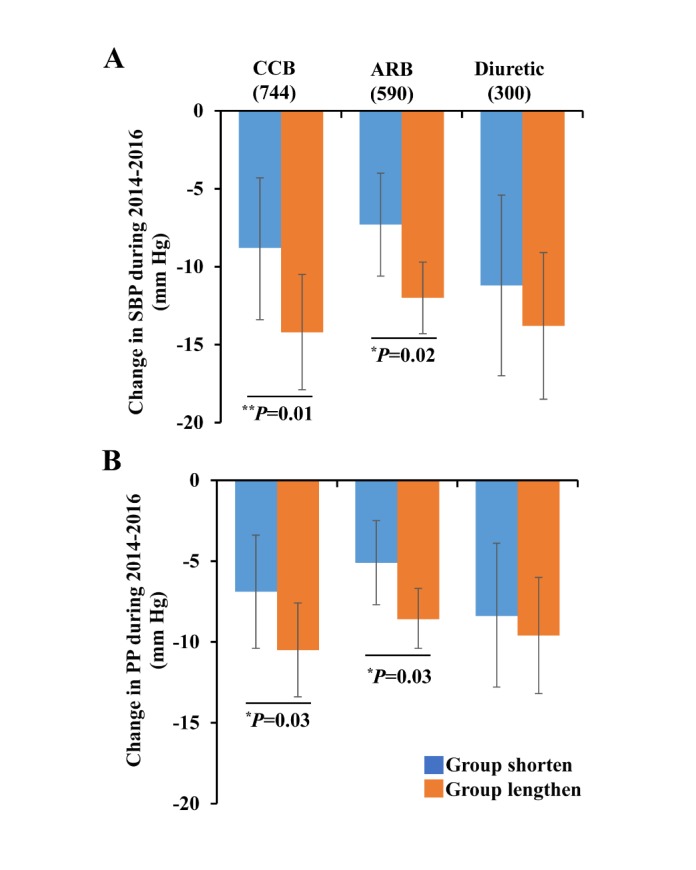
Effects of antihypertensive drugs on blood pressure lowering in the lengthen and shorten groups during 2014-2016. Abbreviations: SBP, systolic blood pressure; PP, pulse pressure; CCB, calcium channel blocker; ARB, angiotensin receptor blocker. ^*^P<0.05, ^**^P<0.01. *P* value and adjusted mean were calculated by generalized linear model adjustment for covariates mentioned in the Figure 2. Error bars indicate 95%CI.

The association between annual telomere attrition rate and blood pressure lowering differed in antihypertensive treatment (Supplemental Table S7). Among patients using the CCB or ARB treatment, those in the lengthen group showed a significantly lower level in ∆SBP and ∆PP than those in the shorten group during 2014-2016. For CCB users, the difference between the lengthen and shorten group was 5.4 mm Hg (95% CI, 1.1-9.7; *P*=0.01) for ∆SBP and 3.6 mm Hg (95% CI, 0.3-6.9; *P*=0.03) for ∆PP; for ARB users, the difference was 4.7 mm Hg (95% CI, 0.6-8.7; P=0.02) for ∆SBP and 3.5 mm Hg (95% CI, 0.3-6.7; P=0.03) for ∆PP (Fig. 3). Consistently, the correlation between telomere attrition and ∆SBP or ∆PP during follow-up was observed in patients treated with CCB and ARB, but not in those with diuretics (Table 3).

**Table 3:** Association of telomere attrition with effects of antihypertensive treatment.

Change in blood	Annual telomere	Model I^*^			Model II^†^		
pressure	attrition rate	*β*	SE	*P *value	*β*	SE	*P *value
CCB therapy (n=744)							
ΔSBP	Shorten (n=251)	Ref.			Ref.		
	Lengthen (n=493)	-4.72	1.95	0.02	-4.73	1.98	0.02
ΔDBP	Shorten (n=251)	Ref.			Ref.		
	Lengthen (n=493)	-1.56	0.98	0.11	-1.62	0.99	0.10
ΔPP	Shorten (n=251)	Ref.			Ref.		
	Lengthen (n=493)	-3.59	1.51	0.02	-3.53	1.53	0.02
ARB therapy (n=590)							
ΔSBP	Shorten (n=192)	Ref.			Ref.		
	Lengthen (n=398)	-5.08	2.13	0.02	-4.75	2.16	0.03
ΔDBP	Shorten (n=192)	Ref.			Ref.		
	Lengthen (n=398)	-1.19	1.08	0.27	-1.31	1.09	0.23
ΔPP	Shorten (n=192)	Ref.			Ref.		
	Lengthen (n=398)	-4.20	1.68	0.01	-3.56	1.70	0.04
Diuretic therapy (n=300)							
ΔSBP	Shorten (n=104)	Ref.			Ref.		
	Lengthen (n=196)	-4.19	2.99	0.16	-2.86	3.06	0.35
ΔDBP	Shorten (n=104)	Ref.			Ref.		
	Lengthen (n=196)	-1.39	1.53	0.36	-1.37	1.53	0.37
ΔPP	Shorten (n=104)	Ref.			Ref.		
	Lengthen (n=196)	-2.10	2.61	0.42	-0.84	2.65	0.75

Abbreviations: CCB, calcium channel blocker; ARB, angiotensin receptor blocker; and the others same as in Table 2. ^*^Model I and ^†^Model II were multiple linear regression analysis for association between annual telomere attrition rate and effect of antihypertensive therapy during 2014-2016, which adjusted for the same covariates described in the footnote of Table 2.

### Association of Telomere Attrition Rate with Cardiovascular Outcomes

During a median follow-up of 2.2 (range 1.5-2.4) years, there were 70 cardiovascular events and 40 stroke events. The results showed that there was no significant correlation between annual telomere attrition rate and cardiovascular events, and the antihypertensive drugs also did not affect this correlation (all *P*>0.05, Supplemental Table S8). In addition, the baseline telomere length as a continuous variable was also not observed to associate with total cardiovascular disease events, and the hazard ratio is 0.76 (95%CI, 0.26-2.24; *P*=0.62).

### DISCUSSION

In this study, we for the first time investigated the relation between annual telomere attrition rate and blood pressure lowering in a longitudinal cohort of 1,108 primary hypertensive patients in China. The key findings showed that telomere lengthening was independently associated with decrease in SBP and pulse pressure during the follow-up period. Compared with hypertensive patients who experienced telomere length shortening, those patients in the lengthen group who experienced an increase of telomere length showed significantly lower levels of SBP and pulse pressure during 2014-2016, and this association was independent of conventional vascular risk factors. Of note, the differences in ∆SBP and ∆PP were more significant in women and patients aged≤60 years. Moreover, the association between annual telomere attrition rate and BP lowering differed in antihypertensive treatment. Among patients using the CCB and ARB therapy, those in the lengthen group showed a significantly lower level in ∆SBP and ∆PP. However, there was no significant correlation between annual telomere attrition rate and incidence of cardiovascular events.

The major strengths of this study are the repeated measurements of blood pressure and telomere attrition via a prospective, longitudinal design with a large sample size as well as the detailed antihypertensive drug records. Several studies have reported cross-sectional associations between shorter leukocyte telomere length with higher levels of blood pressure and pulse pressure [12,15,16,18,27], although there is inconsistence in other study [17]. Here, our data regarding the longitudinal change of telomere length in patients with hypertension provided the evidence that the annual telomere attrition rate was negatively correlated with blood pressure lowering of SBP and pulse pressure during the follow-up, and the association was independent of the stage of baseline blood pressures.

The leukocytes telomere length is highly heterogeneous at birth and changes throughout the lifetime, which is affected by genetic and environmental factors [2,3]. Aging is a major determinant of telomere attrition [4], and as expected, we found that increasing age, as an independent predictor, was inversely associated with both baseline telomere length and follow-up telomere length. In addition, baseline telomere length was observed to independently associate with accelerated telomere attrition, supporting the concept that telomere length shortens much faster at longer baseline telomeres [28].

Several studies have observed that telomere lengthening occurs in approximately 12%-24% of healthy individuals [10,29], but the change of telomere length has not previously been evaluated in patients with hypertension. Given the accumulating pathophysiological burden in chronic illness, it might be expected that telomere shortening would occur in a greater proportion of hypertensive patients. However, almost a third of patients in this study experienced telomere shortening, and more than a half actually lengthened their telomeres during the 2-year follow-up. We did not find that traditional vascular risk factors such as BMI, waist-to-hip ratio, lipid profiles, and serum fasting glucose, were associated with temporal change of leukocytes telomere at follow-up. The regression analysis showed a strong correlation between telomere attrition and baseline telomere length, in which patients who experienced the greater amount of telomere attrition at follow-up were more likely to have a longer baseline telomere length, and in contrast, those who experienced telomere lengthening at follow-up had the shorter baseline telomeres. Supported by the mathematical model of telomere shortening [30], these findings further suggest that there may be a negative feedback regulation of leukocytes telomere length in humans.

Telomere biology is emerging as an important factor in the pathogenesis of hypertension. Low telomerase activity was detected in endothelial progenitor cells (EPC) from hypertensive rats and patients with essential hypertension, which may contribute to premature cell senescence [31]. Moreover, the long-term exposure to risk factors such as oxidative stress and insulin resistance, which are frequently associated with high blood pressure and are known to inhibit telomerase activity and accelerate telomere shortening, may ultimately provoke vascular senescence and promote disease progression by increasing arterial stiffness [32-34]. Okuda et al identified a higher rate of telomere attrition in abdominal aorta, suggesting that telomere shortening may occur in vascular segments susceptible to hemodynamic stress [35]. More basic research is needed to clarify the temporal change of telomerase activity and telomere length in arterial cells from hypertensive patients.

In this study, younger patients (≤60 years) with lengthened telomeres at follow-up had significant blood pressure lowering in ΔSBP, ΔDBP and ΔPP. Elevated blood pressures, considered as clinical markers of large artery stiffness, usually aggravate with aging [36,37]. The increased arterial stiffness can reduce the buffering function of conduit arteries near the heart and increase pulse wave velocity, both of which can increase SBP and pulse pressure [38]. Stiffer arteries make the pressure wave travel faster in the arterial tree, which usually induces systolic rather than diastolic augmentation [39]. Thus, it is possible that the correlation of telomere lengthening with blood pressure lowering among older patients could be attenuated by age-associated arterial stiffness. To date, studies regarding telomeres and cardiovascular diseases among older people also remain inconsistent [40].

A number of studies have found leukocytes telomere length to be longer in women than in men, and the rate of telomere length shortening was slower in women [41-45]. Men tend to have unhealthy life-style behaviors than women [46], and these unhealthy behaviors are associated with reduced telomere length [47,48]. As expected, men in our cohort had greater frequencies of smoking and drinking, a higher level of waist-to-hip ratio, and shorter telomere length at baseline. In addition, estrogen can stimulate telomerase activation by reducing the oxidative stress and thus may be protective against reactive oxygen species damage [49]. These might explain the significant correlation of blood pressure lowering with the lengthened telomere in women, but not in men. For subgroup analysis, this study also had a sufficient power (>90%) to detect blood pressure difference with a two-sided α level of 0.05, based on the difference in means of leukocytes telomere length between the shorten (mean, 0.23; SD, 0.21) and the lengthen group (mean, 0.05; SD, 0.21). Further studies are useful for assessing the effects of gender on telomere maintenance and telomerase in hypertension.

Interestingly, we found that annual telomere attrition rate was associated with the heterogeneity in blood pressure lowering in response to antihypertensive drugs, and telomere lengthening had significantly beneficial effects in ΔSBP and ΔPP among patients treated with CCB or ARB. Antihypertensive drugs may affect cell senescence and telomere length shortening. The CCB has been shown to possess antioxidant properties and reduce the oxidative stress in cardiovascular structures, in which suggested mechanisms are to prevent the inactivation of telomerase and increase eNOS activity during the process of vascular endothelial cell senescence [50]. Data from the Framingham heart study showed that shortened leukocytes telomere length is more frequently presented in hypertensive patients with a higher plasma renin-angiotensin ratio [19]. In addition, animal experiments showed that treatment with an angiotensin II receptor antagonist was associated with reduced oxidative stress and increased telomere length in the spontaneously hypertensive rats [20]. The blood pressure lowering response to antihypertensive drugs in relation to telomere attrition may be in part explained by the link between telomeres and renin-angiotensin system. More examination on serum renin and angiotensin level is helpful to clarify the mechanisms.

Emerging evidence has demonstrated that leukocytes telomere length shortening is associated with higher risk of cardiovascular diseases; however, to date, the results of systematic review and meta-analysis are inconsistent in the literatures on the relation between telomeres and cerebrovascular diseases such as stroke, due to the between-studies heterogeneity. Moreover, these meta-analyses are based on observational studies reflecting the measurement of telomere length at a single time point at baseline. For example, Haycock et al reviewed 24 studies involving 43,725 participants and 8,400 patients with cardiovascular disease (5,566 with coronary heart disease and 2,834 with cerebrovascular disease) and showed that shorter leukocyte telomeres are associated with coronary heart disease, but the association with cerebrovascular disease is less certain [8]. The meta-analysis by D'Mello et al showed that shortened leukocytes telomere length has a significant association with stroke and myocardial infarction, but with a high level of heterogeneity among these studies, indicating that larger, well-designed studies are needed to confirm these findings and explore sources of heterogeneity [51]. A recent meta-analysis demonstrated that increased risk of ischemic stroke is associated with shorter telomere length, but this association is stronger in the retrospective studies and in the Asian population [52].

Longitudinal studies demonstrate that accelerated telomere attrition rate might show stronger association with increased risk of mortality from cardiovascular disease in high-risk population, such as older people aged ≥70 years [10] or patients with stable coronary artery disease [53]. In addition, longitudinal change of leukocytes telomere dynamics contributes to the risk of cardiovascular-related phenotypes, including subclinical atherosclerosis, metabolic syndrome components, and left ventricular mass [11, 54-56]. Notably, the relation between telomere attrition rate and cardiovascular outcomes in hypertensive patients is uncertain.

In the present study, our data showed that there was no significant relationship between telomere attrition rate and incidence of cardiovascular events in hypertensive patients during the follow-up. This might be explained that all hypertensive participants in our cohort had received the antihypertensive treatment, which would reduce the occurrence of cardiovascular events, and thus, the potential effects of accelerated telomere attrition may be attenuated. However, in our study, the limited follow-up period, a relatively smaller person-years, and a short-term exposure duration of telomere attrition, may affect the association between annual telomere attrition rate and cardiovascular events. Further studies with a larger sample size and long-term follow-up will support to identify the relation of telomere attrition with future cardiovascular risk among the hypertensive patients.

Some potential limitations in this study should be mentioned. First, the measurements were restricted to telomere length in circulating leukocytes which do not necessarily reflect telomere attrition in other cell types such as myocardium, endothelium, or atherosclerotic plaque. However, studies by Wilson et al [9] and Zhang et al [26] showed that leukocytes telomere can serve as an appropriate indicator of telomeres in vessel wall and in human atherosclerotic plaques. Second, the quantitative PCR technique employed in this study measures the mean telomere length across all chromosomes present in the patient’s leukocytes. Evidence from animal models suggests that the shortest telomere, rather than the mean telomere length, may be the more important determinant of cell viability and chromosomal stability [57]. Thus, use of mean telomere length could have resulted in a loss of precision with regard to the shortest telomere length in each cell. Third, telomerase activity assays were not performed, which might further clarify the mechanisms of telomere lengthening in hypertensive patients. In addition, it has been reported that statins can attenuate the risk of cardiovascular events conferred by shorter telomeres, whereas this effect is not associated with telomeres lengthening [58]. Given that statin use in this cohort only accounted for 19 (4.9%) in the shorten group and 38 (5.3%) in the lengthen group (*P*=0.89), respectively, their use may not affect the correlation of telomere attrition rate with antihypertensive drugs.

### Conclusion

In summary, this study for the first time showed an association between annual telomere attrition rate and blood pressure lowering in a prospective, longitudinal cohort of primary hypertensive patients. Telomere lengthening was independently associated with decrease in SBP and pulse pressure during the follow-up period of 2 years, and the differences in ∆SBP and ∆PP were more significant in women and younger patients aged≤60 years. Moreover, in patients using CCB or ARB therapy, those having lengthen telomeres showed a significantly lower level of ∆SBP and ∆PP. Our data supported that telomere attrition may provide new sight on telomere biology in clinical intervention for hypertension. Future randomized controlled studies will be helpful to clarify the role of antihypertensive interventions in telomeres lengthening.

## Supplementary Materials

The Supplemenantry data can be found online at: www.aginganddisease.org/EN/10.14336/AD.2019.0721.
