# Supplementary material for: Longitudinal Association of Telomere Attrition with the Effects of Antihypertensive Treatment and Blood Pressure Lowering
Source: Aging Dis. 2019 Jul 21;11(3):494–508. doi: 10.14336/AD.2019.0721 (PMC7220288; doi:10.14336/AD.2019.0721)
Supplement: Supplementary file 1 — The Supplemenantry data can be found online at: www.aginganddisease.org/EN/10.14336/AD.2019.0721. [file AD-11-3-494-s.pdf]

SUPPLEMENTARY DATA

Longitudinal Association of Telomere Attrition with the Effects of Antihypertensive Treatment and Blood Pressure Lowering

Shuyuan Zhang[1], Rongxia Li[1], Yunyun Yang[1], Yu Chen[1], Shujun Yang[1], Jian Li[1], Cunjin Wu[2], Tao Kong[1], Tianlong Liu[1], Jun Cai[3], Li Fu[4], Yanan Zhao[1], Rutai Hui[1], Weili Zhang[1].

[1]State Key Laboratory of Cardiovascular Disease, FuWai Hospital, National Center for Cardiovascular Diseases, Peking Union Medical College & Chinese Academy of Medical Sciences, Beijing, 100037, China
[2] The second hospital of Tianjin Medical university, Tianjin,300211, China
[3] Hypertension Centre, FuWai Hospital, National Center for Cardiovascular Diseases, Peking Union Medical College & Chinese Academy of Medical Sciences, Beijing, 100037, China
[4]Benxi Railway Hospital, Liaoning, 117000, China.

METHODS

Definition of the end-points

The main end-points included the blood pressure change, and the total cardiovascular diseases (defined as nonfatal myocardial infraction [MI] or fatal coronary heart disease [CHD], and stroke). Blood pressure was measured according to the JNC7 recommendation that patients should be seated quietly for at least 5 minutes in a chair, and arm supported at heart level [1]. All patients received at least two measurements and the average recorded. Caffeine, exercise, and smoking should be avoided for at least 30 minutes before measurement.

The diagnosis of myocardial infraction was confirmed if met the criteria of the World Health Organization (WHO) of the symptoms such as retrosternal pain last for at least 30 minutes and not relieve after taking nitroglycerine, and either electrocardiographic abnormal observed or elevated cardiac enzyme levels [2]. Fatal CHD was recognized to have occurred these situations: fatal MI confirmed by hospital records; CHD or MI as the main cause of death on the death certificate [3]. Stroke was diagnosed according to the WHO criteria definition as “the sudden onset of focal (or global) deficit of cerebral function lasting more than 24 hours (except interrupted by surgery or death)” confirmed by a brain computed tomography or magnetic resonance [4].

© 2019. Zhang S et al. Published online at http://www.aginganddisease.org/EN/10.14336/AD.2019.0721

SUPPLEMENTARY DATA

Supplementary Table 1. Comparisons of characteristics between total patients and those providing baseline blood samples in the cohort.

Characteristics	Total patients in the cohort (n=3,671)	Patients with baseline blood samples (n=1,382)	*P* value*
Age, years	61.4 ± 10.6	61.7 ± 9.7	0.39
Men, No. (%)	1,051 (41.3%)	430 (38.8%)	0.16
BMI, kg/m^2	26.0 ± 3.4	26.2 ± 3.1	0.13
Waist-to-hip ratio	0.90 ± 0.06	0.90 ± 0.05	0.91
Systolic BP, mm Hg	155 ± 23	159 ± 21	<0.001
Diastolic BP, mm Hg	88 ± 12	89 ± 12	0.13
Current smoking status, %	19.6	18.8	0.59
Current alcohol intake, %	21.1	18.0	0.04
Medical history, %			
Diabetes mellitus	20.5	23.8	0.03
Cardiovascular diseases	36.7	41.8	<0.001

Abbreviations: BMI, body mass index; BP, blood pressure. Data are given mean ± SD.
***P* value was calculated by the chi-square test for categorical variables and by the *t* test for continuous variables between the total patients and patients with baseline blood samples.

Supplementary Table 2. Characteristics of patients with or without the follow-up blood samples.

Characteristics	Included patients with follow-up blood samples (n=1,108)	Excluded patients without follow-up blood samples (n=274)	*P* value*
Age, years	61.7 ± 9.7	61.0 ± 9.9	0.73
Men, No. (%)	430 (38.8%)	135 (49.3%)	0.002
BMI, kg/m^2	26.2 ± 3.1	26.3 ± 3.4	0.75
Waist-to-hip ratio	0.90 ± 0.05	0.90 ± 0.06	0.60
Systolic BP, mm Hg	160 ± 21	162 ± 21	0.25
Diastolic BP, mm Hg	89 ± 12	90 ± 12	0.21
Fasting serum glucose, mmol/L	6.2 ± 1.7	6.3 ± 1.7	0.51
Lipids, mmol/L			
Total cholesterol	5.7 ± 1.1	5.6 ± 0.9	0.26
Triglycerides	1.6 (1.1-2.3)	1.5 (1.1-2.2)	0.67
HDL-C	1.3 ± 0.3	1.3 ± 0.3	0.77
LDL-C	3.6 ± 0.9	3.5 ± 0.8	0.55
Current smoking status, %	18.8	12.7	0.08
Current alcohol intake, %	18.0	11.5	0.06
Medical history, %			
Diabetes mellitus	23.8	17.5	0.26
Cardiovascular diseases	41.8	37.6	0.34

Abbreviations: BMI, body mass index; BP, blood pressure; HDL-C, high-density lipoprotein cholesterol; LDL-C, low-density lipoprotein cholesterol. Data are given mean ± SD, or median (interquartile range).
***P* value was calculated by the chi-square test for categorical variables, or the *t* test for continuous variables, or the Mann-Whitney *U* test for triglycerides between the included patients and the excluded patients without follow-up blood samples.

© 2019. Zhang S et al. Published online at http://www.aginganddisease.org/EN/10.14336/AD.2019.0721

SUPPLEMENTARY DATA

Supplementary Table 3. Characteristics of patients with or without receiving antihypertensive treatments at recruitment.

Characteristics	Patients with antihypertensive treatment (n=961)	Patients without antihypertensive treatment (n=147)	*P* value*
Age, years	61.9 ± 9.6	60.4 ± 10.6	0.09
Men, No. (%)	363 (37.8%)	67 (45.6%)	0.08
BMI, kg/m^2	26.3 ± 3.1	26.0 ± 3.1	0.33
Waist-to-hip ratio	0.90 ± 0.05	0.90 ± 0.05	0.88
Systolic BP, mm Hg	159 ± 21	160 ± 19	0.77
Diastolic BP, mm Hg	88 ± 12	90 ± 11	0.30
Fasting serum glucose, mmol/L	6.2 ± 1.7	6.3 ± 1.9	0.66
Lipids, mmol/L			
Total cholesterol	5.7 ± 1.1	5.8 ± 1.1	0.82
Triglycerides	1.6 (1.1-2.3)	1.6 (1.1-2.4)	0.70
HDL-C	1.3 ± 0.3	1.3 ± 0.3	0.92
LDL-C	3.6 ± 0.9	3.6 ± 0.9	0.53
Current smoking status, %	18.0	23.8	0.11
Current alcohol intake, %	16.9	25.2	0.06
Medical history, %			
Diabetes mellitus	24.0	22.4	0.76
Cardiovascular diseases	44.4	24.5	<0.001
Baseline telomere length, Lg T/S ratio	0.13 ± 0.22	0.11 ± 0.19	0.46
Annual telomere attrition rate, Lg T/S ratio	0.04 ± 0.2	0.06 ± 0.2	0.28

Abbreviations: BMI, body mass index; BP, blood pressure; HDL-C, high-density lipoprotein cholesterol; LDL-C, low-density lipoprotein cholesterol. Data are given mean ± SD, or median (interquartile range). The leukocytes telomere length was expressed as Lg T/S ratio.

**P* value was calculated by the chi-square test for categorical variables, or the *t* test for continuous variables, or the Mann-Whitney *U* test for triglycerides between the two groups.

© 2019. Zhang S et al. Published online at http://www.aginganddisease.org/EN/10.14336/AD.2019.0721

SUPPLEMENTARY DATA

Supplementary Table 4. Predictors of annual telomere attrition rate as a continuous variable*

	**Annual telomere attrition rate**			
**Variables**	***β* coefficient†**	***P* value†**	**Adjusted *β* coefficient‡**	***P* value‡**
Age, years	-0.04	0.21	-0.12	<0.001
BMI, kg/m^2	-0.005	0.86	0.01	0.67
Waist-to-hip ratio	-0.03	0.40	-0.04	0.17
Systolic BP, mm Hg	0.03	0.32	0.001	0.96
Diastolic BP, mm Hg	0.001	0.98	0.03	0.24
Fasting serum glucose, mmol/L	0.04	0.19	0.001	0.97
Serum creatine, μmol/L	-0.02	0.60	-0.04	0.13
Total cholesterol, mmol/L	0.007	0.83	0.01	0.59
Triglycerides, mmol/L	0.01	0.66	0.02	0.49
HDL-C, mmol/L	0.003	0.92	<0.001	0.99
LDL-C, mmol/L	-0.03	0.35	-0.01	0.64
Current smoking status	-0.01	0.72	0.02	0.49
Current alcohol intake	0.03	0.35	0.02	0.55

Abbreviations: BMI, body mass index; BP, blood pressure; HDL-C, high-density lipoprotein cholesterol; LDL-C, low-density lipoprotein cholesterol.

*Telomere length was presented as Lg T/S ratio.

†The *β* coefficient and *P* value were calculated by linear regression analysis.

‡Adjusted *β* coefficient and *P* value were obtained by linear regression analysis after adjustment for baseline telomere length.

© 2019. Zhang S et al. Published online at http://www.aginganddisease.org/EN/10.14336/AD.2019.0721

SUPPLEMENTARY DATA

Supplementary Table 5. Characteristics of patients with different stages of baseline blood pressure.

Characteristics	Patients with controlled BP (n=144)	Patients with stage 1 hypertension (n=380)	Patients with stage 2 hypertension (n=379)	Patients with stage 3 hypertension (n=205)	*P* value*
Age, years	59.5 ± 8.9	60.5 ± 9.6	62.8 ± 9.9##	63.5 ± 9.7##	<0.001
Men, No. (%)	53 (36.8%)	145 (38.2%)	146 (38.5%)	86 (42.0%)	0.76
BMI, kg/m^2	26.3 ± 3.2	26.2 ± 3.1	26.0 ± 3.1	26.7 ± 3.1	0.05
Waist-to-hip ratio	0.90 ± 0.05	0.90 ± 0.05	0.90 ± 0.06	0.90 ± 0.05	0.91
Systolic BP, mm Hg	129 ± 7	148 ± 7##	166 ± 8##	189 ± 14##	<0.001
Diastolic BP, mm Hg	79 ± 6	86 ± 7##	90 ± 11##	96 ± 16##	<0.001
Fasting serum glucose, mmol/L	5.9 ± 1.3	6.2 ± 1.7	6.2 ± 2.1	6.5 ± 2.1##	0.01
Lipids, mmol/L					
Total cholesterol	5.5 ± 1.0	5.7 ± 1.1	5.7 ± 1.1	5.7 ± 1.1	0.82
Triglycerides	1.5 (1.2-2.2)	1.7 (1.1-2.3)	1.7 (1.1-2.4)	1.6 (1.1-2.5)	0.70
HDL-C	1.3 ± 0.2	1.3 ± 0.3	1.3 ± 0.3	1.4 ± 0.3	0.50
LDL-C	3.5 ± 0.8	3.6 ± 0.9	3.6 ± 0.9	3.6 ± 0.8	0.63
Current smoking status, %	20.8	19.2	16.9	20.0	0.67
Current alcohol intake, %	17.6	16.1	18.6	20.6	0.58
Medical history, %					
Diabetes mellitus	20.1	24.2	23.7	25.9	0.67
Cardiovascular diseases	40.3	38.2	42.2	48.8	0.10
Leukocytes telomere length, Lg T/S ratio					
At baseline	0.17 ± 0.23	0.12 ± 0.21#	0.13 ± 0.22#	0.11 ± 0.21#	0.14
At follow-up	0.26 ± 0.25	0.24 ± 0.27	0.24 ± 0.29	0.24 ± 0.28	0.76

Abbreviations: BMI, body mass index; BP, blood pressure; HDL-C, high-density lipoprotein cholesterol; LDL-C, low-density lipoprotein cholesterol; T, telomere repeat copy; S, single-copy gene globin copy. Data were given as mean ± SD, number (%), or median (interquartile range). The leukocytes telomere length was expressed as Lg T/S ratio.
The controlled BP was defined as BP<140/90 mm Hg, stage 1 as SBP 140-159 mmHg and/or DBP 90-99 mm Hg, stage 2 as SBP 160-179 mmHg and/or DBP 100-109 mm Hg, and stage 3 as SBP ≥180 mm Hg, and/or DBP≥110 mm Hg [5].
**P* values were calculated among the different stages of hypertension by the chi-square test for categorical variables, the ANOVA test for continuous variables, or the Kruskal-Wallis test for triglycerides.
#*P*<0.05, ##*P*<0.001, two-sample comparisons between patients with controlled BP and those with stage 1, or stage 2, or stage 3 hypertension, respectively. The student *t*-test was used for comparison of continuous variables, the chi-square test for categorical variables, and the Mann-Whitney *U* test for triglycerides.

© 2019. Zhang S et al. Published online at http://www.aginganddisease.org/EN/10.14336/AD.2019.0721

SUPPLEMENTARY DATA

Supplementary Table 6. Blood pressure lowering in the lengthen and shorten groups during 2014-2016 stratified by age and gender.

Variables	Mean (95%CI) of BP change*, mm Hg		Adjusted mean (95%CI) of BP change†, mm Hg		Mean difference (95%CI) of BP change between shorten and lengthen group†, mm Hg	*P* value†
	Shorten group	Lengthen group	Shorten group	Lengthen group		
**ΔSBP**						
Total sample (1,108)	-7.9 (-10.2, -5.6)	-11.9 (-13.7, -10.2)	-6.9 (-10.1, -2.9)	-10.2 (-12.8, -7.6)	-3.3 (-6.2, -0.3)	0.03
Men (n=430)	-9.7 (-13.3, -6.0)	-12.1 (-14.9, -9.3)	-9.1 (-13.4, -4.8)	-10.5 (-13.9, -7.0)	-1.4 (-6.0, 3.1)	0.56
Women (n=678)	-6.7 (-9.7, -3.8)	-11.8 (-14.0, -10.2)	-8.1 (-13.2, -3.1)	-12.5 (-17.2, -7.9)	-4.4 (-8.2, -0.6)	0.02
≤60 years (n=506)	-5.4 (-8.5, -2.2)	-12.2 (-14.6, -9.8)	-5.6 (-9.5, -1.7)	-11.3 (-14.3, -8.3)	-5.7 (-9.9,-1.6)	0.007
>60 years (n=602)	-9.9 (-13.1, -6.6)	-11.6 (-14.2, -9.1)	-8.2 (-11.8, -4.6)	-8.9 (-11.8, -5.9)	-0.7 (-4.8, 3.5)	0.75
**ΔDBP**						
Total sample (1,108)	-2.1 (-3.1, -1.0)	-3.1 (-4.0, -2.1)	-1.4 (-3.1, 0.3)	-2.3 (-3.7, -0.9)	-0.9 (-2.5, 0.7)	0.29
Men (n=430)	-2.9 (-4.8, -1.1)	-4.2 (-5.8, -2.7)	-1.9 (-4.3, 0.5)	-2.6 (-4.5, -0.7)	-0.7 (-3.3, 1.8)	0.57
Women (n=678)	-1.5 (-2.8, -0.2)	-2.3 (-3.5, -1.2)	-2.7 (-6.6, 1.2)	-3.9 (-7.8, -0.03)	-1.2 (-3.2, 0.9)	0.27
≤60 years (n=506)	-1.7 (-3.4, -0.1)	-4.1 (-5.5, -2.8)	-1.9 (-4.3, 0.6)	-4.4 (-6.3, -2.5)	-2.5 (-5.0, -0.1)	0.03
>60 years (n=602)	-2.3 (-3.8, -0.9)	-2.1 (-3.3, -0.9)	-1.6 (-3.6, 0.4)	-0.7 (-2.5, 1.0)	0.9 (-1.2, 3.0)	0.42
**ΔPP**						
Total sample (1,108)	-5.8 (-7.7, -3.9)	-8.8 (-10.2, -7.5)	-5.5 (-8.1, -2.9)	-7.9 (-10.0, -5.8)	-2.4 (-4.9, -0.1)	0.04
Men (n=430)	-6.7 (-9.6, -3.8)	-7.8 (-10.0, -5.7)	-7.2 (-10.6, -3.8)	-7.9 (-10.6, -5.2)	-0.7 (-4.3, 2.9)	0.72
Women (n=678)	-5.2 (-7.8, -2.7)	-9.5 (-11.3, -7.7)	-4.9 (-7.8, -2.0)	-8.1 (-10.3, -5.9)	-3.2 (-6.3, -0.1)	0.04
≤60 years (n=506)	-3.6 (-6.2, -1.0)	-8.1 (-9.8, -6.4)	-4.3 (-7.3, -1.3)	-7.4 (-9.7, -5.2)	-3.1 (-6.1, -0.1)	0.04
>60 years (n=602)	-7.6 (-10.3, -4.8)	-9.5 (-11.6, -7.4)	-6.5 (-9.6, -3.4)	-7.9 (-10.4, -5.4)	-1.4 (-4.9, 2.1)	0.43

Abbreviations: SBP, systolic blood pressure; DBP, diastolic blood pressure; PP, pulse pressure (PP=SBP-DBP); CI, confidence interval.

*Mean (95%CI) was calculated by the *t* test.

†Adjusted mean (95%CI) and *P* value were examined by generalized linear model which adjusted for baseline characteristics including age, gender (except for the gender stratification), smoking and alcohol status, medical history, leukocytes telomere length, the stage of baseline blood pressures, and changes in BMI, waist-to-hip, serum fasting glucose, total cholesterol, triglycerides, HDL-C, and LDL-C from 2014 to 2016.

© 2019. Zhang S et al. Published online at http://www.aginganddisease.org/EN/10.14336/AD.2019.0721

SUPPLEMENTARY DATA

Supplementary Table 7. Effects of antihypertensive drugs on BP lowering in the lengthen and shorten groups during 2014-2016.

Variables	Mean (95%CI) of BP change*, mm Hg		Adjusted mean (95%CI) of BP change†, mm Hg		Mean difference (95%CI) of BP change between shorten and lengthen group†, mm Hg	*P* value†
	Shorten group	Lengthen group	Shorten group	Lengthen group		
**ΔSBP**						
CCB (n=744)	-9.9(-12.9, -6.9)	-14.5 (-16.7, -12.3)	-8.8(-13.3, -4.2)	-14.2 (-17.9, -10.5)	-5.4 (-9.7, -1.1)	0.01
ARB (n=590)	-7.4 (-10.7, -4.1)	-12.2 (-14.6, -9.9)	-7.3 (-10.6, -4.0)	-12.0 (-14.3, -9.1)	-4.7 (-8.7, -0.6)	0.02
Diuretic (n=300)	-7.6 (-11.9, -3.2)	-12.1 (-15.6, -8.5)	-11.2 (-17.0, -5.4)	-13.8 (-18.5, -9.1)	-2.6 (-9.2, 4.0)	0.44
**ΔDBP**						
CCB (n=744)	-2.5 (-3.9, -1.0)	-4.1 (-5.3, -3.0)	-1.8 (-4.1, -0.4)	-3.7 (-5.5, -1.9)	-1.9 (-4.0, 0.3)	0.08
ARB (n=590)	-2.0 (-3.6, -0.4)	-3.3 (-4.5, -2.0)	-2.5 (-4.3, -0.8)	-3.9 (-5.2, -2.6)	-1.4 (-3.4, 0.7)	0.20
Diuretic (n=300)	-2.4 (-4.5, -0.3)	-4.2 (-6.1, -2.3)	-2.7 (-5.7, 0.2)	-4.1 (-6.5, -1.8)	-1.4 (-4.7, 1.9)	0.41
**ΔPP**						
CCB (n=744)	-7.4 (-9.9, -4.9)	-10.4 (-12.1, -8.7)	-6.9 (-10.4, -3.4)	-10.5 (-13.4, -7.6)	-3.6 (-6.9, -0.3)	0.03
ARB (n=590)	-5.3 (-8.0, -2.6)	-8.9 (-10.8, -7.1)	-5.1 (-7.7, -2.5)	-8.6 (-10.5, -6.8)	-3.5 (-6.7, -0.3)	0.03
Diuretic (n=300)	-5.1 (-8.6, -1.7)	-7.9 (-10.6, -5.2)	-8.4 (-12.9, -4.0)	-9.6 (-13.2, -6.0)	-1.2 (-6.3, 3.9)	0.65

Abbreviations: SBP, systolic blood pressure; DBP, diastolic blood pressure; PP, pulse pressure (PP=SBP-DBP); CCB, calcium channel blocker; ARB, angiotensin receptor blocker; CI, confidence interval.
*Mean (95%CI) was calculated by the *t* test. †Adjusted mean (95%CI) and *P* value were examined by generalized linear model which adjusted for the covariates mentioned in the footnote of Supplementary Table 6.

© 2019. Zhang S et al. Published online at http://www.aginganddisease.org/EN/10.14336/AD.2019.0721

Supplementary Table 8. The association of annual telomere attrition rate with cardiovascular events during 2014-2016.

Outcomes	The shorten group	The lengthen group	*P* value
**Total patients (n=1,108)**	386	722	
Total CVD, n	30	40	
Person-years	826	1,568	
HR (95% CI), Model I*	1.0	0.73 (0.44-1.19)	0.21
HR (95% CI), Model II†	1.0	0.77 (0.47-1.27)	0.31
Coronary heart diseases, n	15	18	
Person-years	839	1,585	
HR (95% CI), Model I*	1.0	0.49 (0.22-1.09)	0.08
HR (95% CI), Model II†	1.0	0.71 (0.35-1.43)	0.33
Stroke, n	17	23	
Person-years	834	1,576	
HR (95% CI), Model I*	1.0	0.77 (0.39-1.52)	0.45
HR (95% CI), Model II†	1.0	0.75 (0.37-1.49)	0.41
**Patients with CCB therapy (n=781)**	251	493	
Total CVD, n	23	30	
Person-years	537	1,069	
HR (95% CI), Model I*	1.0	0.66 (0.38-1.15)	0.14
HR (95% CI), Model II†	1.0	0.60 (0.34-1.06)	0.08
**Patients with ARB therapy (n=590)**	192	398	
Total CVD, n	9	22	
Person-years	418	863	
HR (95% CI), Model I*	1.0	1.10 (0.46-2.64)	0.84
HR (95% CI), Model II†	1.0	1.30 (0.56-3.02)	0.55
**Patients with diuretic therapy (n=300)**	104	196	
Total CVD, n	8	10	
Person-years	223	426	
HR (95% CI), Model I*	1.0	0.52 (0.19-1.46)	0.21
HR (95% CI), Model II†	1.0	0.56 (0.19-1.63)	0.29

Abbreviations: HR, hazard ratio; CI, confidence interval; CCB, calcium channel blocker; ARB, angiotensin receptor blocker; CVD, cardiovascular disease.

HR (95% CI) and *P* value were examined by the Cox proportional-hazards regression model.

*Model I adjusted for baseline characteristics including age, gender, BMI, waist-to-hip, smoking and alcohol status, medical history, leukocytes telomere length, serum fasting glucose, total cholesterol, triglycerides, HDL-C, and LDL-C. †Model II further adjusted for changes in BMI, serum fasting glucose, total cholesterol, triglycerides, HDL-C, and LDL-C from 2014 to 2016, besides the covariates in Model I.

© 2019. Zhang S et al. Published online at http://www.aginganddisease.org/EN/10.14336/AD.2019.0721

SUPPLEMENTARY DATA

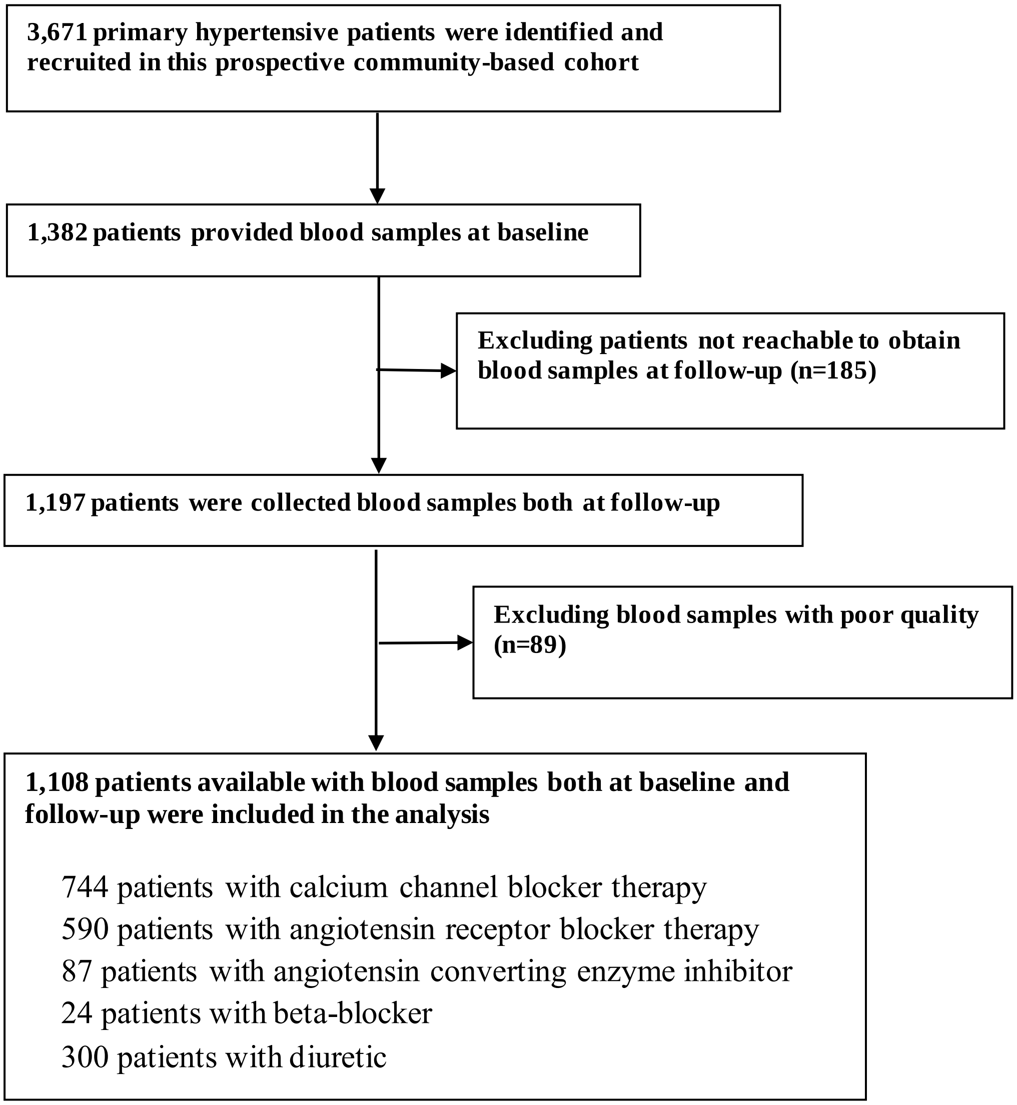

Supplementary Figure 1. Flowchart of the current analysis.

© 2019. Zhang S et al. Published online at http://www.aginganddisease.org/EN/10.14336/AD.2019.0721

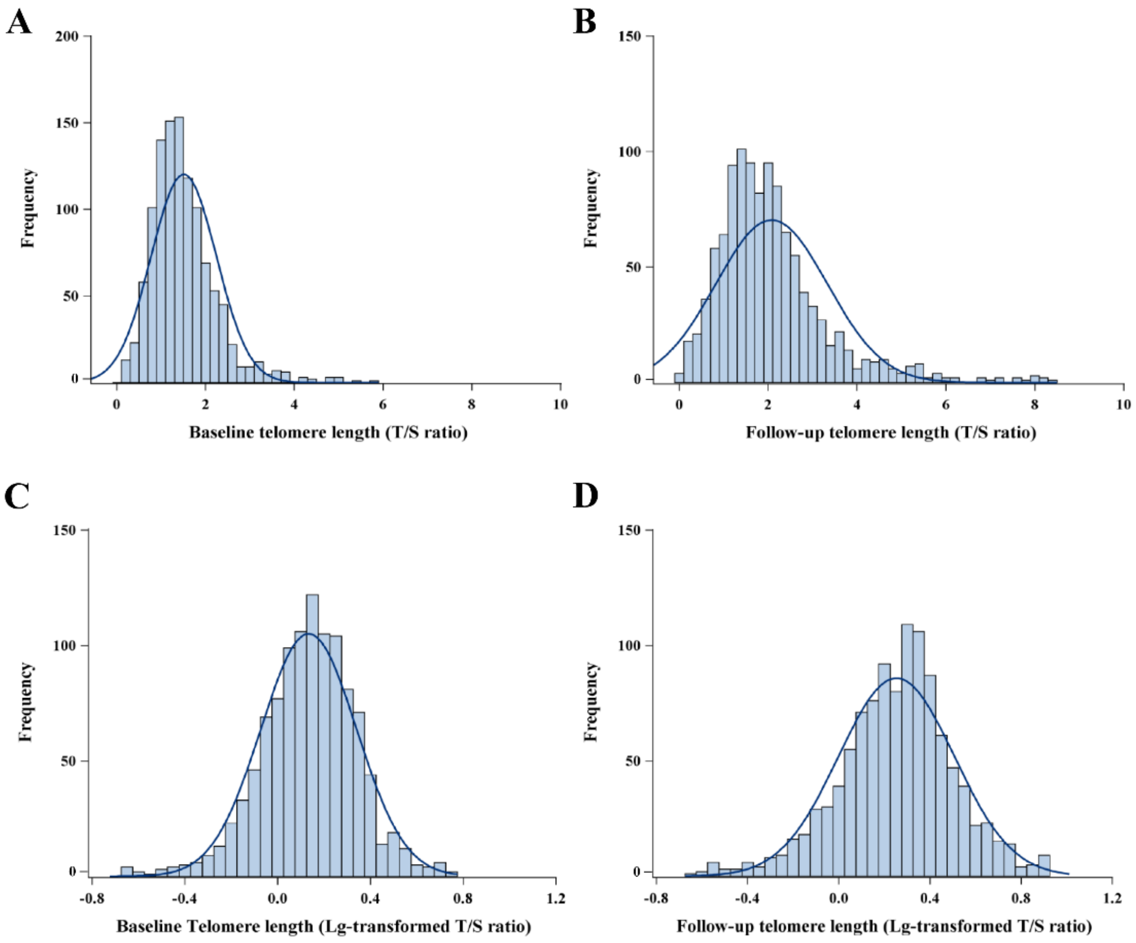

Supplementary Figure 2. The frequency distribution of baseline telomere length and follow-up telomere length in this cohort. (A) Skewed distribution of baseline telomere length of leukocytes; (B) Skewed distribution of follow-up telomere length of leukocytes; (C) Normalized distribution of Lg-transformed baseline telomere length of leukocytes; (D) Normalized distribution of Lg-transformed follow-up telomere length of leukocytes.

© 2019. Zhang S et al. Published online at http://www.aginganddisease.org/EN/10.14336/AD.2019.0721

SUPPLEMENTARY DATA

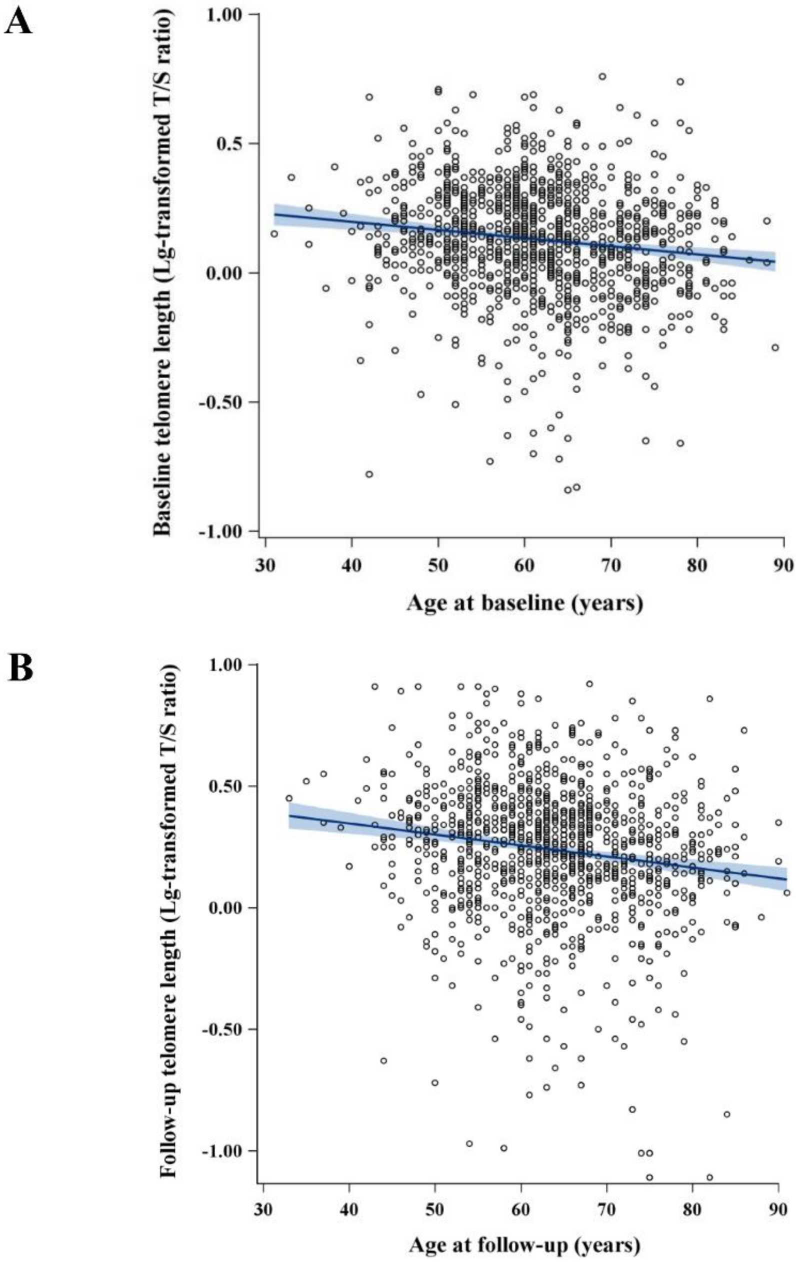

Supplementary Figure 3. The inverse correlations between age and telomere length at baseline and at follow-up. (A) Linear regression between baseline telomere length and age, β value was -0.14 (P<0.001); (B) Linear regression between follow-up telomere length and age, β value was -0.16 (P<0.001).

© 2019. Zhang S et al. Published online at http://www.aginganddisease.org/EN/10.14336/AD.2019.0721

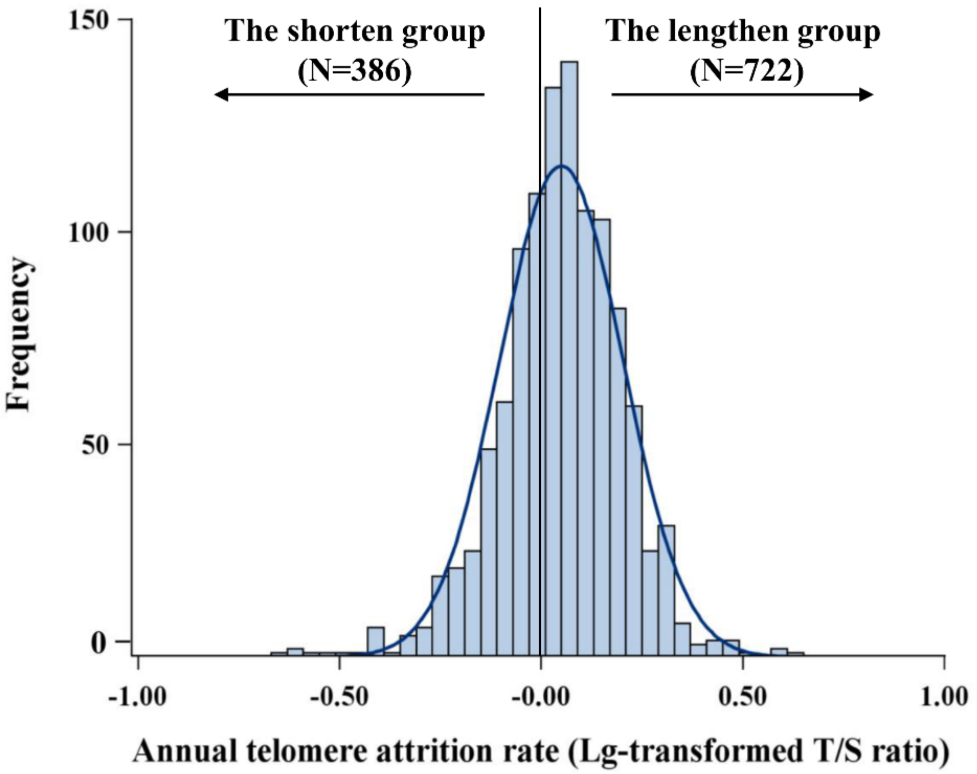

Supplementary Figure 4. The frequency distribution of annual telomere attrition rate during 2014-2016. The annual telomere attrition rate was calculated using the formula: (follow-up telomere length - baseline telomere length)/follow-up years, in which telomere length was transformed as Lg T/S ratio. All participants were categorized into two groups, of those who experienced telomere length shortening (annual rate of telomere attrition <0) were categorized as “shorten group”, and those who experienced an increase of telomere length (annual rate of telomere attrition >0) as “lengthen group”.

© 2019. Zhang S et al. Published online at http://www.aginganddisease.org/EN/10.14336/AD.2019.0721

SUPPLEMENTARY DATA

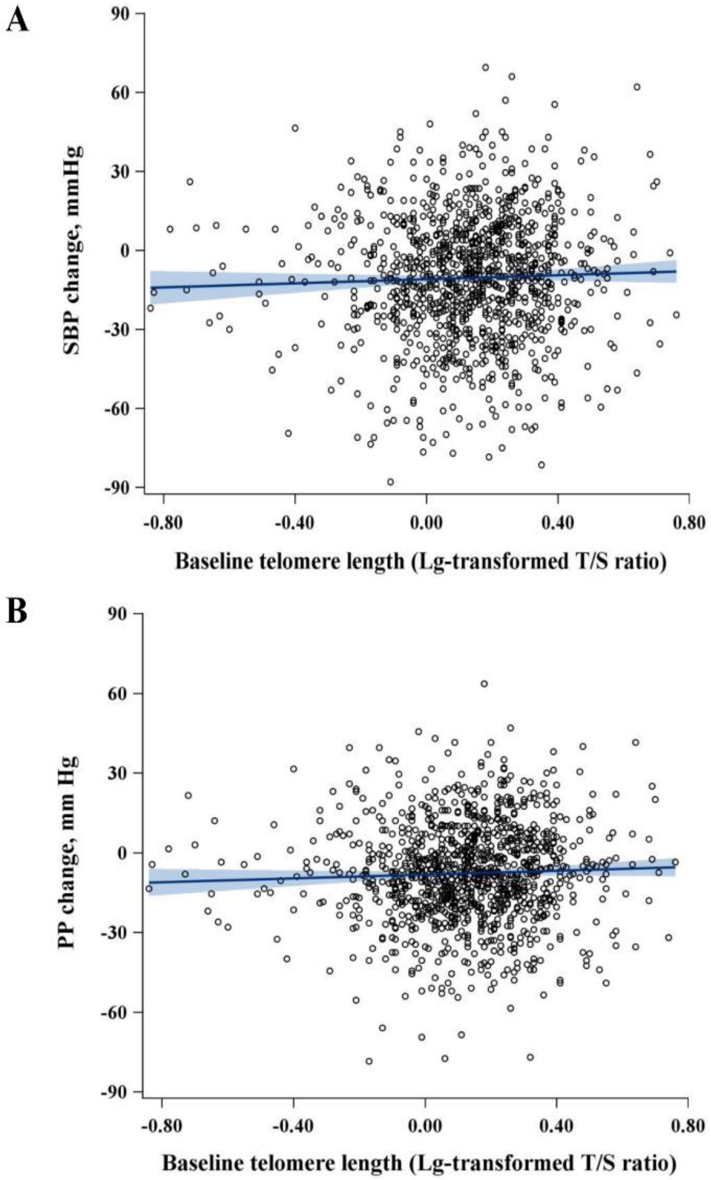

Supplementary Figure 5. No correlations between baseline telomere length and blood pressure change during 2014-2016. Abbreviations: SBP, systolic blood pressure; PP, pulse pressure. (A) Linear regression between baseline telomere length and SBP change, β value was 0.04 (P=0.25); (B) Linear regression between baseline telomere length and PP change, β value was 0.04 (P=0.18).

© 2019. Zhang S et al. Published online at http://www.aginganddisease.org/EN/10.14336/AD.2019.0721

SUPPLEMENTARY DATA

References

[1] National High Blood Pressure Education Program. The Seventh Report of the Joint National Committee on Prevention, Detection, Evaluation, and Treatment of High Blood Pressure. Bethesda (MD): National Heart, Lung, and Blood Institute (US), 2004.
[2] Tunstall-Pedoe H, Kuulasmaa K, Amouyel P, Arveiler D, Rajakangas AM, Pajak A (1994). Myocardial infarction and coronary deaths in the world health organization monica project. Registration procedures, event rates, and case-fatality rates in 38 populations from 21 countries in four continents. Circulation, 90(1):583–612.
[3] Thygesen K, Alpert JS, Jaffe AS, Simoons ML, Chaitman BR, White HD (2012). Third universal definition of myocardial infarction. Glob Heart, 7(4):275-295.
[4] World Health Organization (1989). Recommendations on stroke prevention, diagnosis, and therapy. Report of the WHO task force on stroke and other cerebrovascular disorders. Stroke, 20(10):1407–1431.
[5] Liu LS (2011). 2010 Chinese guidelines for the management of hypertension. Zhonghua Xin Xue Guan Bing Za Zhi, 39(7):579-615.

© 2019. Zhang S et al. Published online at http://www.aginganddisease.org/EN/10.14336/AD.2019.0721
